# Those Vacant Chairs

**Published:** 1897-09

**Authors:** 


THOSE VACANT CHAIRS.—When it is remembered that the medical branch of the University of Texas,—the Texas Medical College at Galveston, is a State institution of Texas, and furthermore,—more's the pity,—that the tax payers pay for the medical education of the students, it seems but reasonable that the medical profession of the State should expect some recognition in the matter of furnishing teachers for said school.

We omit any discussion of the justice or propriety of making property owners pay for a young man's medical education; we accept it as a fact, that they do; and that fact alone should entitle the physicians of the State to a chance, at least, of securing the professorships.

We believe there are men in the State capable of filling any chair in the school with credit and ability. If such is the case, the Regents will, no doubt, in time, have an opportunity of ascertaining,—for there will be applications from Texas physicians, we know.

The physicians of Texas are called upon to aid the school by sending their students there,—to their home school. The profession of Texas feel an interest in the school and a pride in its success, and have responded to the call with a cordial good will; but should the policy continue to prevail of selecting the teachers wholly or in greater part from New England or Europe, no one should complain, or be surprised if they withhold or withdraw their support, and advise those studying under them to matriculate elsewhere. It is the very best course that could be pursued to alienate the element on whom the State and the management of the University rely for support and co-operation.

Already there has been furnished room for complaint on this head. In the original organization of the medical faculty the Regents went to Europe for two teachers, to Pennsylvania for one, and to Mexico for one; all good men and able teachers beyond a doubt, but the question has been asked; was it necessary to go away from Texas to find teaching talent for a school supported by Texas taxpayers? We know that there has been a good deal of soreness over this fact, and now that two Texas physicians who having served the school to the best of their ability several years, and so far as we have been informed, with fair average ability, at least, are virtually kicked out by the regents, for we learn that it was only by the timely intervention of a collegue, that the chair of one of the gentlemen—a distinguished, able and popular physician, was not declared vacant,—in his absence; without so much as the courtesy of a request for his resignation, the profession of the State have little encouragement to hope to supply their places by Texas recruits.

We believe that, considering the foregoing facts, it will be for the best interests of the school for the Regents to fill these chairs by appointment from the profession at home; assuming, of course, that we have the requisite talent here; for if the successors of Drs. Clopton and West are chosen from abroad, the school will no longer have the good will of the profession, and at next session of the legislature we will find that a certain element already not very kindly disposed will renew their efforts to protect the tax payers, as they say, from the burden of setting men's sons up in business. We have heard it argued in legislative circles that it would be as just to tax the people to raise the wherewith for a stock of merchandise as to tax them to equip a young man to practice medicine, or foı any other livelihood.

We do not mean to make any threat; far from it. But we predict that unless the Texas physicians are recognized in the bestowal of these professorships, there will be a falling off in classes—a falling off in good will and co-operation on the part of the doctors, and a kick will come that will startle the most sanguine of the friends of the school. It will be remembered that it was by the skin of their teeth the management secured any appropriation last session. If the four or five thousand physicians of Texas are ignored in this matter and are turned against the school, it is exceedingly questionable whether the next legislature can be prevailed on to make any appropriation for the school at all.

